# A Comparative Study of Asymptomatic Malaria in a Forest Rural and Depleted Forest Urban Setting during a Low Malaria Transmission and COVID-19 Pandemic Period

**DOI:** 10.1155/2022/2545830

**Published:** 2022-10-15

**Authors:** Clarisse E. Mbah, Lum A. Ambe, Adela Ngwewondo, Elvis B. Kidzeru, Lilian Akwah, Celestin Mountchissi, Mohamadou Mansour, Edward N. Sahfe, Rene´ Kamgang, Lucia Nkengazong

**Affiliations:** ^1^Institute of Medical Research and Medicinal Plants Studies (IMPM), Centre for Research on Health and Priority Pathologies (CRSPP), P.O. Box 13033 Yaoundé, Cameroon; ^2^Department of Microbiology and Parasitology, Faculty of Science, University of Buea, P.O. Box 63 Buea, Cameroon; ^3^Hair and Skin Research Laboratory, Division of Dermatology, Department of Medicine, Faculty of Health Sciences and Groote Schuur Hospital, University of Cape Town, Cape Town, South Africa; ^4^National Health Laboratory Service (NHLS) Tygerberg Laboratories, Medical Microbiology, Immunology and Virology laboratories, Division of Medical Microbiology and Immunology, Department of Pathology, Stellenbosch University, South Africa; ^5^University of Yaoundé 1, Cameroon, Faculty of Science, Microbiology Department, Cameroon

## Abstract

The global malaria morbidity and mortality witnessed an increase from 2019 to 2020 partly due to disruptions in control programs' activities imposed by the COVID-19 pandemic. Therefore, there is still a significant burden of malaria in Cameroon which needs attention from all fronts to attain elimination goals. It is normally expected that a typical forest ecology that has undergone urbanization and subjected to high rates of ecological instabilities should also have a shift from characteristic perennial malaria transmission and a shift in the type of malaria endemicity plaguing such distorted forest ecology. In this observational comparative study, we randomly enrolled participants from rural and urban settings of a forest zone during a low malaria transmission period, which coincided with the onset of COVID-19 pandemic. An optimized structured questionnaire was employed, to collect socio-demographic data and associated risk factors. The CareStart™ Malaria HRP2 antigen test was performed on participants from both settings to determine the prevalence of community asymptomatic malaria. Of 307 participants, 188 (61.0%) were from the rural, while 119 (38.8%) from the urban community. The overall prevalence of asymptomatic malaria (27.0%) detected *Plasmodium falciparum* antigen in 83 participants. The urban community's prevalence was 4.2% (5 positives) while the rural community's was 41.5% (78 positives). In simple logistic regression models, rural forest community and farm around the house were statistically significant predictors of testing positive (coefficient 2.8, 95% CI 1.8-3.7, *p* value<0.001) and (coefficient 3.1, 95% CI 1.1-5.1, *p* value =0.003), respectively. In the multivariate model, the strongest predictor of testing positive was living in a rural community, with *p* < 0.001 and odds ratio of 10.9 (95% CI, 3.8-31.8). These results indicate that during a low transmission period, the prevalence of asymptomatic malaria differs between depleted urban and rural forested settings, suggesting a need for strategic target intervention for the control of asymptomatic malaria.

## 1. Introduction

The World Health Organization (WHO) estimated malaria cases to have increased from 213 to 241 million, and deaths from 534 000 to 627 000 globally between 2019 and 2020 [1]. Of these, 96% occurred in sub-Saharan Africa, where it is the second leading cause of death, with pregnant women and children being the most affected [2].

Despite the scale-up of malaria control interventions and substantial decline of global malaria morbidity and mortality experienced in 2019 before the COVID-19 pandemic in 2020, it is rather unfortunate that there is still a significant burden of the disease, caused especially by disruptions due to COVID-19 in 2020 [1], the period in which this study was carried out.

Generally, people living in a forest area are more at risk of having malaria as the forest ecosystem are well known to support transmission, therefore significantly contributing to the global disease burden [3].

The ‘forest ecotype' is defined by UNESCO as terrain with a tree canopy cover of more

than 10% and an area of more than 0.5 hectares, including natural forests and plantations [4] with a minimum tree height of 5 m, including coffee, rubber, cork oak, and fruit tree plantations, wind break, and shelter belts more than 20 m width [5].

Malaria transmission is perennial in a typical forest ecotype [6] making its control in such an ecotype a major challenge [3], even though it is estimated that almost half the malaria risk occurs among people living in forested areas [7, 8].

A typical forest ecology that has undergone urbanization and subjected to much ecological instability (caused by deforestation, poor housing, and lack of proper sanitation and drainage systems) should experience a shift from this characteristic perennial transmission and its unique endemicity.

Understanding the malaria transmission triangle (host-parasite-environment) in distorted forest ecology is pivotal in the epidemiology and control of the disease. There have been numerous studies on the spread of malaria, but it is still unclear how urbanization of a forest ecotype influences human transmission of the illness. According to research that examined the entomological transmission of the illness, the rise in cases of malaria and other vector-borne illnesses in Africa, particularly in Cameroon, may have been caused by a high rate of ecological instability [9–12]. However, this has not been examined with focus on the contribution of human activities to malaria transmission even though rural forested communities near their densely populated urban/peri-urban counterpart could serve as hidden foci of drug and insecticide resistance with inadequate surveillance and control measures [3].

Asymptomatic malaria refers to the presence of malaria parasites without symptoms within a period of two days and at the time of testing [13, 14]. There is evidence that parasites of asymptomatic carriers are more infectious than symptomatic carriers given that their continuous exposure to mosquito bites fuels the transmission pipeline, hence more studies on asymptomatic malaria are needed [15]. There is still a long way for malaria elimination to be realized in some endemic forest communities, especially in Africa. This is partly because asymptomatic parasite carriage remains the bedrock that fuels transmission and is not specifically targeted by most interventions. This therefore, is responsible for propagating the parasite population from one transmission season to the next [16, 17].

It is therefore of major public health importance to determine how urbanization impacts changes in disease transmission, which influences differences in the disease pattern among asymptomatic carriers. This is relevant in detecting communities most affected by malaria, for adequate interventions. In this COVID-19 era, it is particularly important that all efforts be put in place to reduce the current malaria burden which was heightened by the effects of the pandemic.

We therefore aimed at assessing the variation of human malaria (detected using *P. falciparum* Ag RDT strips) among asymptomatic carriers between a rural forested ecotype, and a distorted forest urban community during a low malaria transmission and COVID-19 period, as there is limited information to understand how human activity through the urbanization of a forest ecotype affects malaria transmission.

## 2. Materials and Methods

### 2.1. Study Area

The study was conducted in the Centre Region of Cameroon, one of the most densely populated regions, with a population of about 4.09 million [18]. The Centre Region is part of the southern plateau of Cameroon and is located at the heart of the dense rainforest. The mean altitude of this broad plateau is 600-700 m and it is almost dominated by 900 m hills separated from deep valleys. Its climate is cool and mild with high and mean temperatures of 25°C, rainfall ranging from 1500 to 2000 mm, and mean precipitations of about 1572 mm [19, 20]. The dominant vegetation is a dense tropical forested environment which gradually becomes or transforms into savannah towards the North [18, 19, 21]. It is a hot spot of forest depletion where one can observe severe ecosystem degradations [22]. The entire region was once heavily forested, however in the current state, exploitation of wood land, construction, and urbanization has thinned out the forest ecology resulting in very little virgin land in the region. The region is made up of 10 divisions characterized by rural, urban, and peri-urban dwellers.

The Mbam-et-Inoubou Division is home to the little virgin land in the region and was selected as the study site for the rural forested ecotype community. Enrollment to the study was done at the heart of the forest settlement, precisely at the Donenkeng (11°17 Par 4°43), Niatchotta (11°38 Par 4° 24), and Ngommo (11°17 Par 4°46) villages. Fishing, sand excavation, and agriculture are the main activities of inhabitants in this area.

Participants of the urban depleted forest ecotype community were enrolled from the Mfoundi Division which is the main cosmopolitan city of the region. It is home to people of various origins and religious denominations. The division has characteristics of an urban settlement which includes several paved national roads and railway lines connecting with neighboring regions. It also has an International Airport, one of two in the country, and the administrative buildings to all ministries in the country. All these make it to have a built-up surface and an appropriate site.

### 2.2. Climatic Condition

The Centre Region experiences four equatorial seasons, alternating between rainy and dry periods. The dry season begins the year, running from December to May, and later from July to October. Then the rainy season, which lasts from May to June and subsequently ends the year from October to November [23].

### 2.3. Study Period

The end of September and October was selected for the assessment of the human transmission among asymptomatic carriers. This period has been reported to have zero infective bites per man per month in urban forest settings [9] and a low malaria transmission in a forest zone [24], which was also during the start of the COVID-19 pandemic.

### 2.4. Ethical Considerations

The study was conducted under the ethical clearance No. 2020/02/1214/CE/CNERSH/SP conferred by the Cameroon National Ethics Committee for Research on Human Health (CNERSH) on 20 February 2020. Written and verbal consent was obtained from the participants, and they were assured of the anonymity and confidentiality of their information. There was no financial benefit for participating in the research and participation was on a voluntary basis. Participants were free to withdraw from the study at any given point.

### 2.5. Data Collection Method

This was a comparative cross-sectional community study. In both the rural and urban communities, an information note containing a brief background, objective, procedures, voluntary nature of participation, declarations of anonymity, and confidentiality was provided. Due to the difference in the structure of the rural versus urban communities, the methods used for mobilization were slightly different. In the rural community, after prior notification and confirmation from the district and traditional authorities, the community was mobilized for two weeks by using community healthcare workers through a focal person to recruit participants. The screening for malaria was done during three consecutive days. For the urban community, participants were randomly selected in the community for three consecutive days. Consent and assent were obtained before participants were enrolled for the study. All consented and assented participants were interviewed with a questionnaire and blood samples collected thereafter.

### 2.6. Inclusion Criteria

All those who voluntarily gave consent and assent to participation in the study signed the consent or assent form. To avoid imported malaria cases from either urban or rural settings or vice versa, only those who had lived in the community for at least a year or were born in the community were included.

### 2.7. Exclusion Criteria

Those who refused to give consent or assent by signing the respective forms were excluded. Also, all clinically ill individuals were excluded. Clinically ill individuals are those who had some malaria related symptoms (temperature ≥37.5°C, chills, severe malaise, headache, or vomiting).

### 2.8. Questionnaire Administration

An optimized questionnaire was administered. Questions included demographic information (age, gender, and occupation), how long they had stayed in their community and the presence or absence of farm around the house to assess the vector proximity to the population and breeding sites. On the questionnaire, participants were required to tick ‘yes' or ‘no' to the question ‘do you have farm or bushes around the house?', to determine if they had farms or bushes at proximity to the house.

### 2.9. Parasitological Analysis

A point of care rapid diagnostic malaria parasitological test was performed using the CareStart™ Malaria *P. falciparum* (HRP2) Ag RDT strip (Access Bio, Inc., at Somerset NJ, USA) according to manufacturer's instructions. This is an antigenic immunochromatographic method which detects the *P. falciparum* Histidine Rich Protein 2 (PfHRP-2). The Ag RDT test is recommended for establishing a malaria case with satisfactory sensitivity and specificity [25, 26]. To maximize our chances of diagnosing most of the asymptomatic cases, we decided to employ the CareStart test, which is more sensitive than microscopy [27–29], easy to handle in our setting, fast and does not require specific infrastructure or training [30]. The kit was labelled with the respective sample code and for each enrolled participant, the left index finger was disinfected with an alcohol swab, and whole blood sample was collected by piercing with a lancet. The first drop of whole blood was wiped out with a dry cotton swab and 5 *μ*L of the subsequent flow was collected as specimen directly onto the sample well of the device. Two drops of lysis buffer solution (60 *μ*L) were added to the buffer well to lyse the cells, release the antigen, and facilitate antibody recognition. The test results were read after 20 minutes and interpreted as positive or negative. The presence of two lines indicated a positive result for *P. falciparum*, while the presence of a single line, indicated a negative result. Asymptomatic malaria carriers were defined as those who did not present any overt signs and symptoms of the disease but tested positive for malaria with the RDT test strip.

### 2.10. Statistical Analysis

Data entry, coding, and corrections were done using Microsoft® Excel. Occupation was categorized in to three categories including skilled (accountants, pastors, contractors, researchers, laboratory technicians, entrepreneur etc.), semi-skilled (barbers, builders, mechanic, business, carpenters), and others (sand miners, farmers, students, housewives, guards, jobless). Statistical Product and Service Solutions (SPSS) version 20 was used to compute descriptive statistics including Fisher's exact, Chi-squares, and Rank-sum tests to assess for significant associations between variables. STATA version 11 was used to perform inferential statistics including univariate and multiple regression models.

## 3. Results and Discussion

### 3.1. Results

A total of 307 participants were enrolled in this study with median (interquartile range, IQR) age 30 (12 to 50) years. Rural participants were significantly older than those in the urban setting. Amongst the 307 participants, 187 (60.9%) were females and 120 (39.1%) males, 188 (61.0%) were from the rural community while 119 (38.8%) from the urban community. The average time, median (IQR) in years, that participants enrolled had stayed in the rural community was 16.5 (12-40) years and that of the urban dwellers was 11 (4-35) years, and participants in the rural community had stayed significantly longer in their community than had those in the urban setting (Table 1).

The overall prevalence of malaria in our study was 27.0% (83 positive *P. falciparum* Ag tests). The urban community's prevalence was 4.2% (5 positive *P. falciparum* Ag tests), while the rural community's prevalence was 41.5% (78 positive *P. falciparum* Ag tests) indicating a 9.9 times significantly higher prevalence in the rural compared to that of the urban setting (Supplementary Table 1). Other important statistically significant associations observed between variables and *P. falciparum* (HRP2) Ag RDT strip test status included farm around the house and occupation, all with *p* values ≤0.001.

### 3.2. Univariate Analysis

Simple logistic regression was performed to assess the impact of each predictor variable on the *P. falciparum* (HRP2) Ag RDT strip test status, and the likelihood that respondents will test positive. Each predictor variable was added in the univariate analysis and the impact assessed separately, one at a time. The rural dwellers were more likely to test positive for malaria than urban dwellers (coefficient 2.8, 95% CI 1.8-3.7) (Table 2). Also, those with farms around the house were predictive of being infected with *P. falciparum* (coefficient 3.1, 95% CI 1.1-5.1) (Table 2). With respect to occupation, there was a positive association with positive *P. falciparum* (HRP2) Ag RDT strip test (coefficient 1.0, 95% CI 0.3-1.7) (Table 2).

Given that the variable occupation was grouped as earlier described in the statistical analysis section into three categories including being skilled, semi-skilled and others, those in the category “others” recorded the highest frequency with detectable *P. falciparum* (HRP2) Ag [n =76, (30.3%)]. This could be driving the positive significant association observed between occupation and testing positive for *P. falciparum* malaria.

After adjusting for a priori confounders (predictor variables) including community, age, gender, farm around the house, and occupation, bold text represents statistical significance. Predictor variables were added and evaluated as independent models in the univariate analysis and the impact assessed one at a time.

### 3.3. Multivariate Analyses

Multiple logistic regression analysis was performed using all statistically significant variables in the univariate logistic regression models, to assess the impact on the *P. falciparum* (HRP2) Ag RDT strip test status and the likelihood that the respondent will test positive. The models contained three predictor (independent) variables including community, farm/bushes around the house and occupation. Variables were added in a stepwise manner. In the full model containing all predictors the rural community was a statistically significant predictor of *P. falciparum* (HRP2) Ag detection with a *p* value<0.001 (coefficient 2.385, 95% CI 1.313-3.458) (Table 3). Meanwhile, only the ‘type of community' was significant in the model among the predictor variables.

This therefore indicated that the ‘type of community' was a stronger and an independent predictor variable for testing positive for malaria as compared to having a farm around the house or having a particular occupation. Our data shows that the strongest predictor of a positive *P. falciparum* malaria test is living in a rural community (coefficient 2.385, 95% CI 1.313-3.458), with an odds ratio (OR) of 10.862 (95% CI, 3.718-31.739) units higher with all other predictors held constant (Table 3). This indicates that participants who stayed in a rural community were more than 10 times likely to have malaria than those who did not.

Further, when community was removed from the model, farm around the house became a significant predictor of a positive *P. falciparum* malaria RDT test with *p* value = 0.006 (coefficient 2.804, 95% CI 0.796-4.813), after correcting for occupation in model 3. This indicates that the odds of having positive *P. falciparum* malaria RDT test is more likely to be 16.516 (95% CI, 2.216-123.097) units higher with all other predictors held constant (Table 3). Those without farm around the house are therefore less likely to have a positive *P. falciparum* malaria test to the same degree. Occupation was also a statistically significant predictor of a positive *P. falciparum* malaria RDT test with *p* value = 0.028 (coefficient 0.768, 95% CI 0.081-1.455). The type of occupation had higher odds of predicting a positive *P. falciparum* malaria RDT test, with OR of 2.156 (95% CI, 1.085-4.285) units higher with all other predictors held constant (Table 3).

Multiple logistic regression models predicting *Plasmodium* RDT, after adjusting for confounders (predictor variables) that were statistically significant predictors of *Plasmodium* RDT positive test included community, farm around the house, and occupation. Bold text represents statistical significance. Each predictor variable was added in the multivariate analysis in a stepwise manner and the impact assessed one at a time [Model 1: Community and Farm around the house, Model 2: Community, Farm around the house and Occupation, and Model 3: Farm around the house and profession (Removing Community form the model)].

## 4. Discussion

The aim of this study was to compare asymptomatic malaria between a rural and urban setting in a forest ecotype using the RDT strip and to understand predictors of malaria transmission in these settings. We obtained an overall prevalence of 27%, similar to the 22.9% reported in 2018 in Cameroon [31]. The prevalence of asymptomatic malaria in the rural community was 41.5% while that in the urban counterpart was 4.2%. In a simple logistic regression model, rural forest community, farm around the house, and various occupations were statistically significant predictors of a positive malaria test. In the multivariate models, after adding all the statistically significant independent variables from the univariate models, the strongest predictor of a positive test was living in a rural community. We were able to show that, the prevalence of asymptomatic malaria differs between urban and rural settings in a forest zone.

This study was carried out at the heart of the COVID-19 pandemic in 2020. This period had been demonstrated as a low transmission period in the forest zone [10]. It is worth noting that this is contrary to known facts that human transmission in many places peaks during and proceeding the rainy season [32]. The COVID-19 pandemic could be an explanation of this observation, as activities of malaria control programs were disrupted during this period, encouraging reservoirs for parasites to develop within the communities. Similar reports of high asymptomatic malaria has been reported in Central African Republic during this period of our study [33], indicating that the pandemic had an immense effect on malaria control. This was recently explained by WHO world malaria report which recorded a 12% increase of malaria cases from 2019 to 2020, of which an estimated 47000 (68%) of the additional 69000 malaria deaths were due to disruptions during the COVID-19 pandemic [1]. With the pandemic contributing only partially to this increase therefore, other factors were also responsible for the increase in cases which needs to be identified and tackled. The presence of asymptomatic malaria is a threat to disease control, as it fuels disease recrudescence and the development of drug resistance [34].

Our findings of 41.5% prevalence of asymptomatic malaria in the rural community alone indicates that the rural forested ecotype communities contribute more to the overall prevalence of asymptomatic malaria than the urban counterpart. This observation is different from the low prevalence of 6.8% in rural Ethiopia, where microscopy was the method of diagnosis for asymptomatic malaria [35]. We determined 9.9 times more asymptomatic malaria prevalence in the rural forested community than that of the depleted forest urban community (4.2%). Therefore, looking globally at the overall prevalence in Cameroon can be misleading on the actual contribution of the various subgroups (rural and urban settings), of which knowledge of the difference in prevalence between the rural and urban settings zooms in better on the contribution of each counterpart to the overall burden. This knowledge would inform and enable control programs to rationally distribute resources according to the needs of the various regions to meet their demands amid scarcity in resources [26]. The low prevalence (4.1%) of asymptomatic malaria recorded in the depleted forest urban community indicates an impact of urbanization on the transmission of malaria in the forest ecotype. This is similar to 4.8% prevalence of asymptomatic malaria in a 2022 study in urban Ethiopia using the RDT test strip [33, 36].

Our results are contrary to previous predictions that deforestation in Central Africa and tropical America might increase malaria burden [26, 37]. Entomological explanations could hold that urban areas are generally regarded as unsuitable for the propagation of the vectors as deforestation causes instability of the vector environment thereby enhancing mosquito-human malaria transmission. Furthermore, socio-economic factors including easy access to antimalaria therapy and healthcare facilities, higher economic and social status, and better housing conditions are certainly more important for urban dwellers than with their rural counterpart.

Having a Farm/bush around the house was found by our study to have an association to testing positive for malaria (*p* = 0.003). The higher prevalence of asymptomatic malaria in the rural forest community might be attributed to certain human practices like overnight stays within forests to cultivate and collect forest produce, hunting, wide-open household construction [3], and a farm/bush around the house. Those who had a farm/bush around the house were more likely to be infected with *Plasmodium* spp. This finding, though not new, indicates that vegetation near human habitation increases the population of forest-dwelling malaria vectors and thus results in an increase in entomological inoculation rate/transmission [3] and hence increased occurrence of human malaria as indicated by our study.

With respect to occupation, those in the category ‘other', comprised of sand miners, farmers, students, housewives, guards, etc., had the highest frequency of a positive malaria test and could be driving the positive significant association with testing positive for malaria. We found the association of the various occupations and a positive malaria test as an interesting public health intervention strategy to control asymptomatic malaria. Behavioral changes, including wearing of protective clothing at various occupational sites in rural forested communities could be considered as an intervention strategy.

Factors accounting for 9.9 times difference of asymptomatic malaria between the rural and urban forest zones warrant investigation to fully understand how urbanization of forest ecology affects the dynamics of malaria transmission. The difference in prevalence in the forested urban and rural community might not be identical to that of the other ecological rural and urban settings and warrant further investigation given that Cameroon is ecologically very diverse. Our study used the more sensitive and specific [25, 26] Ag RDT strip to demonstrate that forest rural communities have a pool of asymptomatic cases possibly serving as reservoirs for the malaria parasite. This tool can be implemented for use before the distribution of antimalarial chemotherapeutic agents for the prevention of malaria while targeting specific malaria high risk groups. This would include current WHO-recommended malaria chemo preventive therapies [26, 38, 39]; intermittent preventive treatment during pregnancy (IPTp), coadministration of intermittent preventive treatment to infants (IPTi), and seasonal malaria chemoprevention (SMC) in infants, as well as considerations for chemotherapeutic prevention of asymptomatic carriers [40, 41]. There is evidence [17], and we have shown that asymptomatic malaria infections play an important role in malaria transmission. Interventions to target the parasite reservoirs are necessary to attain malaria elimination in both low (urban) and high (rural) transmission communities [9]. Even though WHO recommends that all suspected cases should be tested, and only confirmed malaria cases treated [24, 25], this strategy might be limited in our context and most of sub-Saharan Africa given that most people living in the endemic regions barely seek healthcare services. Those who self-medicate are excluded and in turn fuel the resistance to existing antimalarial medicines. Generally, rural areas face several challenges to accessing healthcare, some of which are infrastructural difficulties involved with accessing the hinterlands for interventions by control programs, lack of electricity, manpower as well as the appropriate skills in carrying out the standard microscopy tests. To solve this problem, we therefore suggest an evaluation of the efficacy of training community workers and/or families on the use of Ag RDT strip to improve on the diagnosis before treatment. This will in turn help to curb drug resistance and improve on the identification of asymptomatic cases as a community-based intervention strategy especially at this time of the COVID-19 pandemic era.

As a limitation of the study, we acknowledge the lack of data on household characteristics in rural vs. urban settlements which might have an impact on differences in level of transmission recorded. We also think that due to this limitation, it makes it difficult to establish or conclude if variation in the infections observed were because of the presence and/or absence of forest, or to a broader extent due to rural versus urban settlement, shifting seasons as a result of climate change or interruption of intervention programs due to the COVID-19 pandemic. This provides an opportunity for future research whose findings would be far-reaching.

## 5. Conclusions and Recommendations

Our study showed that asymptomatic malaria prevalence was 9.9 times higher in the forested rural community than in the distorted forest urban community during the low malaria transmission season. This implies that the impact of malaria varies depending on the community's ecological context. So, resources should be directed towards communities with higher malaria transmission rates. Also, we showed that living in a rural community and having a farm/bush around the house may influence the prevalence of malaria. Thus, this could be beneficial for public health interventions to reduce asymptomatic malaria in the rural community. Therefore, there is a need to improve and design new intervention strategies because recent efforts to reduce malaria morbidity and mortality are yet to realize their full impact in our communities. Scientific and theoretical evidence suggests that the current set of interventions will not be sufficient to eradicate malaria from many areas in sub-Saharan Africa [16]. Hence, more needs to be done to enforce interventions such as test-and-treat programs by providing training to community workers on the use of RDT, so that they are equipped to test and treat asymptomatic cases and in so doing, target the parasite reservoir.

In addition, with the recent WHO approval of a malaria vaccine RTS, S/AS01, known by the brand name Mosquirix™, as of October 2021, governments should be encouraged to start working towards a vaccination strategy for our communities [42, 43]. This will in the long-term help to achieve malaria elimination in both low- and high-transmission areas.

## Figures and Tables

**Table 1:** Demographics of enrolled participants in the rural and urban communities.

Variables	Category	Total participants, *n* = 307 (%)	Rural, *n* = 188 (%)	Urban, *n* = 119 (%)
Age (year)	*n* *Median (IQR)*	307 (100) *30 (12-50)*	188 (61.2) *38.5 (21-54.5)*	119 (38.8) *17 (8-34)*
	0-4	19 (6.2)	6 (3.1)	13 (10.9)
	5-9	29 (9.4)	10 (5.3)	19 (16)
	10-14	41(13.4)	19 (10.1)	22 (18.5)
	≥15	218 (71.0)	153 (81.4)	65 (54.6)
Gender	Male	120 (39.1)	73 (38.8)	47 (39.5)
	Female	187 (60.9)	115 (61.2)	72 (60.5)
Occupation	Skilled	25 (8.1)	2 (1.1)	23 (19.3)
	Semi-skilled	31 (10.1)	11 (5.9)	20 (16.8)
	Others	251 (81.8)	175 (93.1)	76 (63.9)
Time spent in the village (year)	*n* *Median (IQR)*	307 (100) *39 (12-32)*	188 (61.2) *16.5 (12-40)*	119 (38.8) *11 (4-35)*
	0-10	87 (28.3)	39 (20.7)	48 (40.3)
	11-20	78 (25.4)	61 (32.4)	17 (14.3)
	21-50	106 (34.5)	69 (36.7)	37 (31.1)
	51-90	36 (11.7)	19 (10.1)	17 (14.3)
Farm around the house	Yes	259 (84.4)	188 (100)	71 (59.7)
	No	48 (15.6)	0 (0)	48 (40.3)
Sleep under net	Yes	67 (64.4)	0 (0)	67 (64.4)
	No	37 (35.6)	0 (0)	37 (35.6)
	Unknowns	203 (66.1)	—	—

^∗∗^Fisher's exact unless otherwise noted, ^∗^Rank sum test, ^$^Chi-square tests.

**Table 2:** Simple logistic regression models predicting *P. falciparum* (HRP2) Ag RDT strip test status.

Variables	Category	N	Univariate models	
			Coef. (95% CI)	p-value
Community^a^	Rural	188	2.782 (1.842-3.724)	**<0.001**
	Urban	119		
Age (years)	Median (IQR) 30 (12-50) years	307	-0.005 (-0.017-0.007)	0.409
Gender^b^	Female	187	0.176 (-0.336-0.688)	0.501
	Male	120		
Farm around the house^c^	Yes	259	3.059 (1.061-5.058)	**0.003**
	No	47		
Occupation^d^ (others = 3)	Skilled	25	1.023 (0.337-1.709)	**0.003**
	Semi- skilled	31		
	Other	251		
Sleep under net^e^	Yes	67	0.827 (-1.403-3.056)	0.467
	No	37		

N: Number of observations, Coef: Coefficient, 95% CI: 95% confidence interval, ^a^Rural is the reference category, ^b^Male is the reference category, ^c^Having farm around the house is the reference category, ^d^Others (sand miners, farmers, students, housewives, guards, jobless combined to form one category) is the reference category, ^e^Sleeping under net is the reference category.

**Table 3:** Multiple logistic regression models predicting *Plasmodium* RDT after adjusting for confounders (predictor variables).

Variable	Multivariate models		
	Coef. (95% CI)	OR (95% CI)	*P* value
1) Community^a^ Farm around the house^b^	2.475 (1.425-3.524) 1.01 (-1.213-3.233)	11.877 (4.158-33.931) 2.746 (0.297-25.367)	**<0.001** 0.373
2) Community^a^ Farm around the house^b^ Occupation^c^	2.385 (1.313-3.458) 0.959 (-1.270-3.188) 0.268 (-0.515-1.05)	10.862 (3.718-31.739) 2.609 (0.281-24.234) 1.307 (0.598-2.858)	**<0.001** 0.399 0.502
3) Farm around the house^b^ Occupation^c^	2.804 (0.796-4.813) 0.768 (0.081-1.455)	16.516 (2.216-123.097) 2.156 (1.085-4.285)	**0.006** **0.028**

Coef.: Coefficient; OR: Odds Ratio; 95% CI: 95% confidence interval; ^a^Rural is the reference category; ^b^Having farm around the house is the reference category; ^c^Others (sand miners, farmers, students, housewives, guards, jobless combined to form one category) is the reference category.

## Data Availability

The data used to support the findings of this study were generated from the study and are included within the article and supplementary materials.

## Supplementary Materials

Supplementary Materials Supplementary Table 1 shows the association between variables and CareStart™ Malaria *P. falciparum* (HRP2) Ag. RDT strip test status.
