# Supplementary material for: A Comparative Study of Asymptomatic Malaria in a Forest Rural and Depleted Forest Urban Setting during a Low Malaria Transmission and COVID-19 Pandemic Period
Source: Biomed Res Int. 2022 Oct 15;2022:2545830. doi: 10.1155/2022/2545830 (PMC9587908; doi:10.1155/2022/2545830)
Supplement: Supplementary Materials — Supplementary Table 1 shows the association between variables and CareStart™ Malaria P. falciparum (HRP2) Ag. RDT strip test status. [file 2545830.f1.docx]

# BioMed Research International

**A Comparative Study of Asymptomatic Malaria in a Forest Rural and Depleted Forest Urban Setting During a Low Malaria Transmission and COVID-19 Pandemic Period.**

Clarisse E. Mbah,^1,2,#^ Lum A. Ambe,^1,2,#^ Ngwewondo Adela,^1^ Elvis B. Kidzeru,^1,3,4^ Akwah Lilian,^1,5^ Mountchissi Celestin,^1^ Mohamadou Mansour,^1,5^ Edward N. Sahfe,^1^ Rene´ Kamgang,^1^ Lucia Nkengazong^1^

^1^Institute of Medical Research and Medicinal Plants Studies (IMPM), Centre for Research on Health and Priority Pathologies (CRSPP), P.O Box 13033 Yaoundé, Cameroon.

^2^Departments of Microbiology and Parasitology, Faculty of Science, University of Buea, P.O Box 63 Buea, Cameroon.

^3^Hair and Skin Research Laboratory, Division of Dermatology, Department of Medicine, Faculty of Health Sciences and Groote Schuur Hospital, University of Cape Town, Cape Town, South Africa.

^4^National Health Laboratory Service (NHLS) Tygerberg Laboratories, Medical Microbiology, Immunology and Virology laboratories, Division of Medical Microbiology and Immunology, Department of Pathology, Stellenbosch University.

^5^University of Yaoundé 1, Cameroon, Faculty of Science, Microbiology Department, Cameroon

#These authors are first authors of this work

Correspondence should be addressed to Lum A. Ambe; [lumabienwiambe@yahoo.com](mailto:lumabienwiambe@yahoo.com)

**Supplementary Table 1: Association between variables and CareStart^TM^ Malaria *P. falciparum* (HRP2) Ag RDT strip test status. Bold text represents statistical significance.**

| Variables | Category | RDT Positive (%) | RDT Negative (%) | p-value** |
| --- | --- | --- | --- | --- |
| Community | *Total* | *83 (27.0)* | *224 (73.0)* | **<0.001** |
|  | Rural | 78 (41.5) | 110 (58.5) |  |
|  | Urban | 5 (4.2) | 114 (95.8) |  |
| Age (years) | 0-4 | 5 (26.3) | 14 (73.7) | 0.543* |
|  | 5-9 | 7 (24.1) | 22 (75.9) |  |
|  | 10-14 | 14 (34.1) | 27 (65.9) |  |
|  | ≥15 | 57 (26.1) | 161 (73.9 |  |
| Gender | Female | 48 (25.7) | 139 (74.3) | 0.501^$^ |
|  | Male | 35 | 85 |  |
| Farm around the house | Yes | 82 (31.7) | 177 (68.3) | **<0.001** |
|  | No | 1 (2.1) | 46 (97.9) |  |
| Occupation | Professional | 0 (0.0) | 25 (100) | **0.001** |
|  | Skilled | 7 (22.6) | 24 (77.4) |  |
|  | Other | 76 (30.3) | 175 (69.7) |  |
| Sleep under net | Yes | 4 (6.0) | 63 (94.0) | 0.653 |
|  | No | 1 (2.7) | 36 (97.3) |  |

**Fisher’s exact unless otherwise noted, *Rank sum test, ^$^Chi square tests
